# KLHL5 Is a Prognostic-Related Biomarker and Correlated With Immune Infiltrates in Gastric Cancer

**DOI:** 10.3389/fmolb.2020.599110

**Published:** 2020-12-10

**Authors:** Qiulin Wu, Guobing Yin, Jinwei Lei, Jiao Tian, Ailin Lan, Shengchun Liu

**Affiliations:** ^1^Department of Endocrine and Breast Surgery, The First Affiliated Hospital of Chongqing Medical University, Chongqing, China; ^2^Department of Breast and Thyroid Surgery, Second Affiliated Hospital of Chongqing Medical University, Chongqing, China

**Keywords:** gastric cancer, KLHL5, prognosis, immune infiltration, markers

## Abstract

**Background:** KLHL5 (Kelch Like Family Member 5) is differentially expressed in gastric cancer, but its correlation with prognosis and functioning mechanism in gastric cancer remain unclear.

**Methods:** The Oncomine database and TIMER were employed to appraise the KLHL5 expression in a variety of cancers. The correlation between KLHL5 expression and patient prognosis was extracted from the Kaplan–Meier plotter, GEPIA, and PrognoScan database. Then the relationship between KLHL5 expression and inflammatory infiltrate profiles was inquired by TIMER. Finally, GEPIA and TIMER were explored for the correlative significance between KLHL5 expression and immune cell–related marker sets.

**Results:** KLHL5 was found to be differentially expressed and correlated with clinical outcomes in several types of cancers in the TCGA database. Especially, KLHL5 mRNA expression was upregulated and correlated with poorer overall survival and progression-free survival in gastric cancer. Moreover, elevated KLHL5 expression was significantly related with patient node stage, infiltration level, and expression of multiple immune marker sets.

**Conclusions:** These results implicate that KLHL5 expression is closely linked with patient clinical outcomes and the microenvironmental infiltration level in different neoplasms. This indicates that KLHL5 is a modulator in infiltrate recruitment, shaping the landscape of immune cell infiltration. Thus, it represents an eligible prognostic predictor for gastric malignancy.

## Introduction

Gastric cancer is the leading drive of cancer-related mortality in humans, and poor prognosis of this disease is partly attributed to metastasis (Siegel et al., 2017). In recent years, reports of immunological regulation in the progression of gastric cancer are accumulating (Wang et al., 2014; Bizzaro et al., 2018; Murata, 2018); intrinsically, multiple immunotherapies have been proposed as potential treatments for this malignancy (Moehler et al., 2016; Procaccio et al., 2017). In non-small cell lung carcinoma, immunoregulatory agents targeting CTLA4, PD-1, or PD-L1 have showed promising inhibitory effect (Osmani et al., 2018), but in gastric cancer, anti-CTLA4 drug tremelimumab failed to bring anticipated results in clinical setting (Ralph et al., 2010), and PD-1 and PD-L1 inhibitors failed to make for complete response in most advanced gastric cancer and colorectal cancer patients (Le et al., 2015; Muro et al., 2016; Overman et al., 2017). Immune infiltrates in tumors like tumor-associated macrophages and tumor-infiltrating neutrophils are significantly relevant to patient prognosis and efficacy of therapeutics (Waniczek et al., 2017; Zhang et al., 2018). Thus, there is an urgent necessity for the insight into the immune patterns and their underlying mechanism to help in identifying novel agents in the treatment of gastric malignancy.

The Kelch-like (KLHL) gene family is a group of evolutionarily conserved genes encoding proteins containing BTB domains and Kelch motifs (Dhanoa et al., 2013). Within these structures, the BTB takes part in recruitment degradation substrates to E3 ubiquitin ligase complexes by working as a bridge between the target recognition protein and the scaffold protein Cullin-3 (CUL3) (Perez-Torrado et al., 2006), and the Kelch motif is associated with actin kinetics by forming different types of binding sites (Adams et al., 2000). From previous studies, members of KLHL family are noted for their roles in cell signaling mechanisms including ubiquitination, actin dynamics, and cell cycle pathways (Perez-Torrado et al., 2006; Liu et al., 2020; Zhao et al., 2020).

KLHLs are critical regulators in many different signaling pathways. KLHL6 was reported to be involved in B-lymphocyte antigen receptor signaling, and deficiency of KLHL6 induced failure of B-cell germinal center proliferation in mice (Kroll et al., 2005). KLHL12 inhibited the activation of Wnt/beta-Catenin pathway by degrading Disheveled, a core canonical and non-canonical intermediate in this signaling (Angers et al., 2006). KEAP1 (KLHL19) was found to suppress the activity of anti-oxidative stress pathways by inhibiting the transactivation of cytoprotective transcription factor Nrf2 (Itoh et al., 1999). KLHL21 negatively regulated NF-kB signaling by blocking the function of IKKβ (Mei et al., 2016). KLHL22 modulated ubiquitination of mitotic kinase Polo-like kinase 1 (PLK1), thus helped in governing G2/M checkpoints in the cell cycle (Metzger et al., 2013). These examples demonstrate that KLHLs play important roles and potentially impact on prognosis in cancer patients, even though many of them remain unstudied within literature.

In this study, we selected KLHL5 to investigate its potential as a biomarker of prognosis and explore its underlying mechanism in gastric cancer. KLHL5 is differentially expressed in many benign and malignant lesions, and the complete function of it remains unclear. KLHL5 was overexpressed in ovary, adrenal gland, and thyroid, but less abundant in trachea, prostate, testis, lymph node, and spinal cord tissues (Wang et al., 2001). Kim et al. (2018) found that KLHL5 was significantly upregulated in peritoneal seeding metastasis than paired primary colorectal cancer cells by whole-exome sequencing and microarray analysis. Schleifer et al. (2018) reported that KLHL5 knockdown inhibited proliferation in ovarian adenocarcinoma and renal carcinoma cell lines, and sensitized tumor cells to anticancer agents. KLHL5 was also found to be facilitating ubiquitination of Sphingosine kinase 1 (SK1), a central molecule involved in cell death (Powell et al., 2019).

In our study, we aim to envision the landscape of KLHL5 and its relationship with patient prognosis in cancers, and try to shed some light on the underlying mechanism of KLHL5 functions in cancers, particularly in gastric adenocarcinoma.

## Materials and Methods

### Oncomine Database Analysis

The Oncomine database compiles a colossal load of transcriptome data of over 18,000 genes covering major cancer types in humans (Rhodes et al., 2007). We assessed the mRNA level of KLHL5 in varied tumor types with this database on https://www.oncomine.org/resource/login.html. The threshold was set as follows: *P* < 0.01, fold change ≥2, and gene rank on the top 10%.

### PrognoScan Database Analysis

PrognoScan database comprises a big compendium of published tumor microarray data sets and several helpful modules for mining the relationship between gene profile and patient outcomes across varied malignancies (Mizuno et al., 2009). The relationship between KLHL5 and patient clinical outcomes in multiple cancer types was examined with the PrognoScan database on http://dna00.bio.kyutech.ac.jp/PrognoScan/index.html.

### Kaplan–Meier Plotter Database Analysis

The Kaplan–Meier plotter incorporates transcriptome data of over 50,000 genes with accompanying clinical outcomes extracted from 6,000+ mammary, 2,100+ ovarian, 3,000+ lung, and 1,000+ gastric cancer samples (Lánczky et al., 2016), and it also offers different analyzing tools to adjust related parameters to examine the correlation between expressed genes and cancer patient prognosis in certain conditions. Thus, this database on http://kmplot.com/analysis/ was surveyed to investigate the association between KLHL5 and patient survival in gastric, mammary, ovarian, and lung neoplasm, respectively.

### TIMER Database Analysis

TIMER offers another novel means for systematical analysis of tumor-infiltrating cells throughout different cancers, and more than a toolbox, it contains pre-calculated statistics of immune infiltrates subsets in more than 10,000 tumor cohorts from 32 cancer types (Li et al., 2017). We explored this database to estimate the expression of KLHL5 and its correlation with immune infiltration status and patterns in multiple types of cancers with Diff Exp and Gene module, respectively. Then Kaplan–Meier curve analyses were employed with Survival module to illustrate the cumulative survival as the outcome of gene expression or inflammatory infiltration. Finally, the relationship between KLHL5 and gene markers associated with particular immune cells was also validated with Correlation module on https://cistrome.shinyapps.io/timer/.

### GEPIA Database Analysis

GEPIA integrates large loads of microarray data sets of gene expression profiles in over 9,000 tumors and more than 8,000 normal control tissues based on the TCGA and the GTEx database accompanying patients' clinical information (Tang et al., 2017). It also provides various modules to facilitate data mining. We surveyed the correlative ratio and significance between KLHL5 and prognosis in different cancers with Survival plots module, then inquired the relationship of KLHL5 with particular gene markers associated with immunological infiltrates in tumors with Correlation module via this database on http://gepia.cancer-pku.cn/.

### Statistical Analysis

The expression profile of KLHL5 from Oncomine database is displayed with fold change no < 2, *P-*value smaller than 0.01, and gene rank of the top 10%. The plots from Kaplan–Meier plotter and GEPIA are presented with HR and log-rank *P-*value, and the curves from PrognoScan are exhibited similarly. Results from TIMER are displayed with cor, *P*, or log-rank *P. P* or log-rank *P*-value smaller than 0.05 is regarded statistically significant in the aforementioned results. In terms of correlative degree between variables, Spearman's correlation was applied and the absolute value of cor was used to determine the strength of correlation: 0.00–0.19 as “very weak,” 0.20–0.39 as “weak,” 0.40–0.59 as “moderate,” 0.60–0.79 as “strong,” and 0.80–1.0 as “very strong.” *P*-values under 0.05 were considered to be the cutoff value of significance.

## Results

### Landscape of KLHL5 Expression in Different Cancers

**Figure 1:**
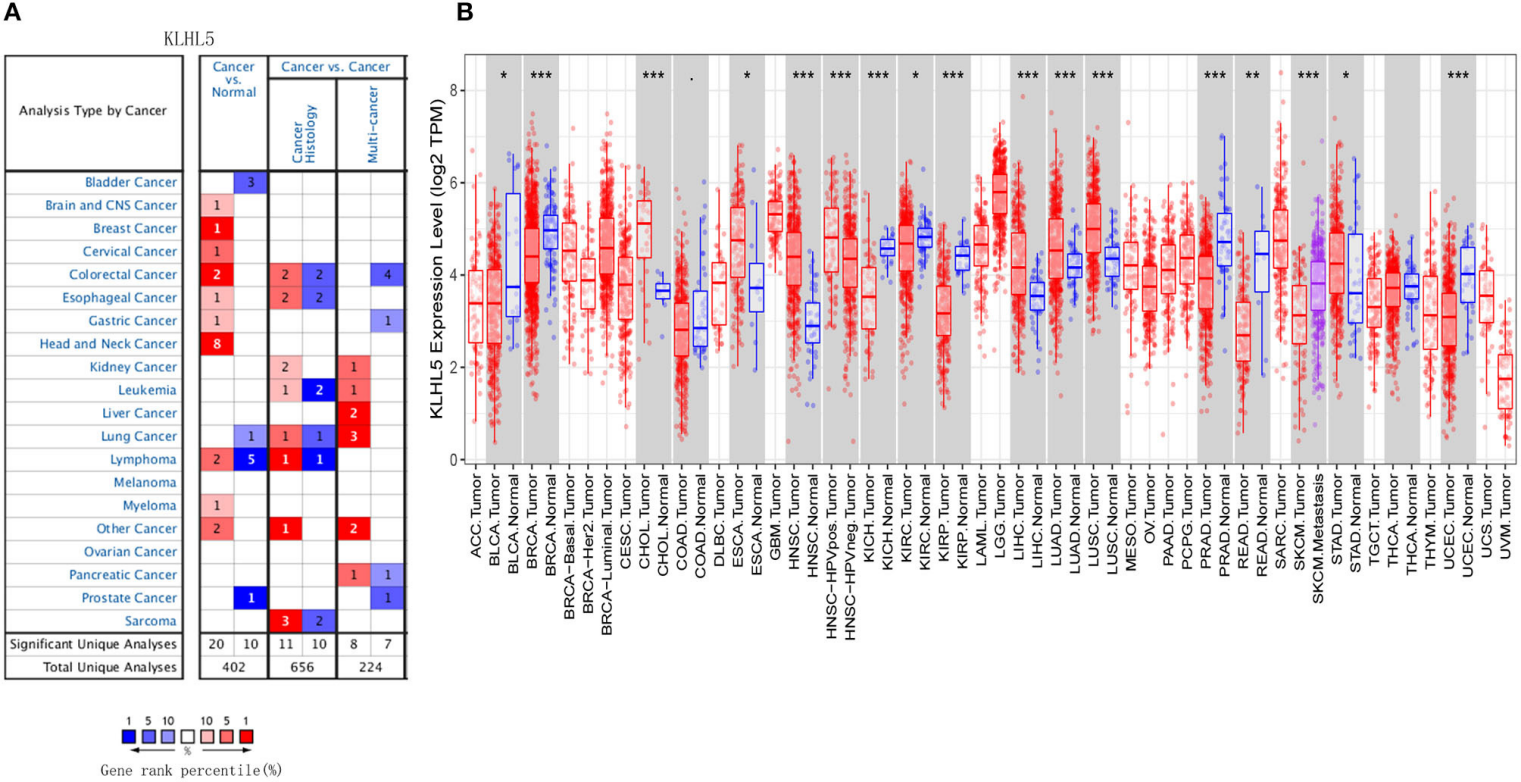
Full landscape of KLHL5 expression in different malignancies. **(A)** Differentially expressed KLHL5 in cancer tissues and normal controls in Oncomine database. **(B)** KLHL5 expression profile in multiple cancer types in TIMER database. *p < 0.05, **p < 0.01, and ***p < 0.001.

First, the Oncomine was explored for illustrating the full landscape of KLHL5 in different malignant and adjacent benign tissues, and it revealed that KLHL5 was upregulated in mammary, cervical, head and neck, brain, colorectal, esophageal, lymphoma, and gastric cancer tissues in comparison with their normal controls (Figure 1A). In addition, its expression in lung, bladder, lymphoma, and prostate cancer was shown to be downregulated in multiple data sets. Complete profile of KLHL5 expression in different tumors is collated in Supplementary Table 1.

Next, we evaluated the differentially expressed level of KLHL5 in different malignancies in TCGA. Results of TIMER analysis showed that the level of KLHL5 was significantly elevated in CHOL (cholangiocarcinoma), ESCA (esophageal carcinoma), HNSC (head and neck squamous cell carcinoma), LIHC (liver hepatocellular carcinoma), LUAD (lung adenocarcinoma), LUSC (lung squamous cell carcinoma), STAD (Stomach adenocarcinoma) compared with adjacent normal samples. In contrast, its expression was less abundant in BLCA (bladder urothelial carcinoma), BRCA (breast invasive carcinoma), KICH (kidney chromophobe), KIRC (kidney renal clear cell carcinoma), KIRP (kidney renal papillary cell carcinoma), PRAD (prostate adenocarcinoma), READ (rectum adenocarcinoma), and UCEC (uterine corpus endometrial carcinoma) tissues in comparison with adjacent normal controls (Figure 1B).

### KLHL5 Expression Correlates With Prognosis in Tumors

**Figure 2:**
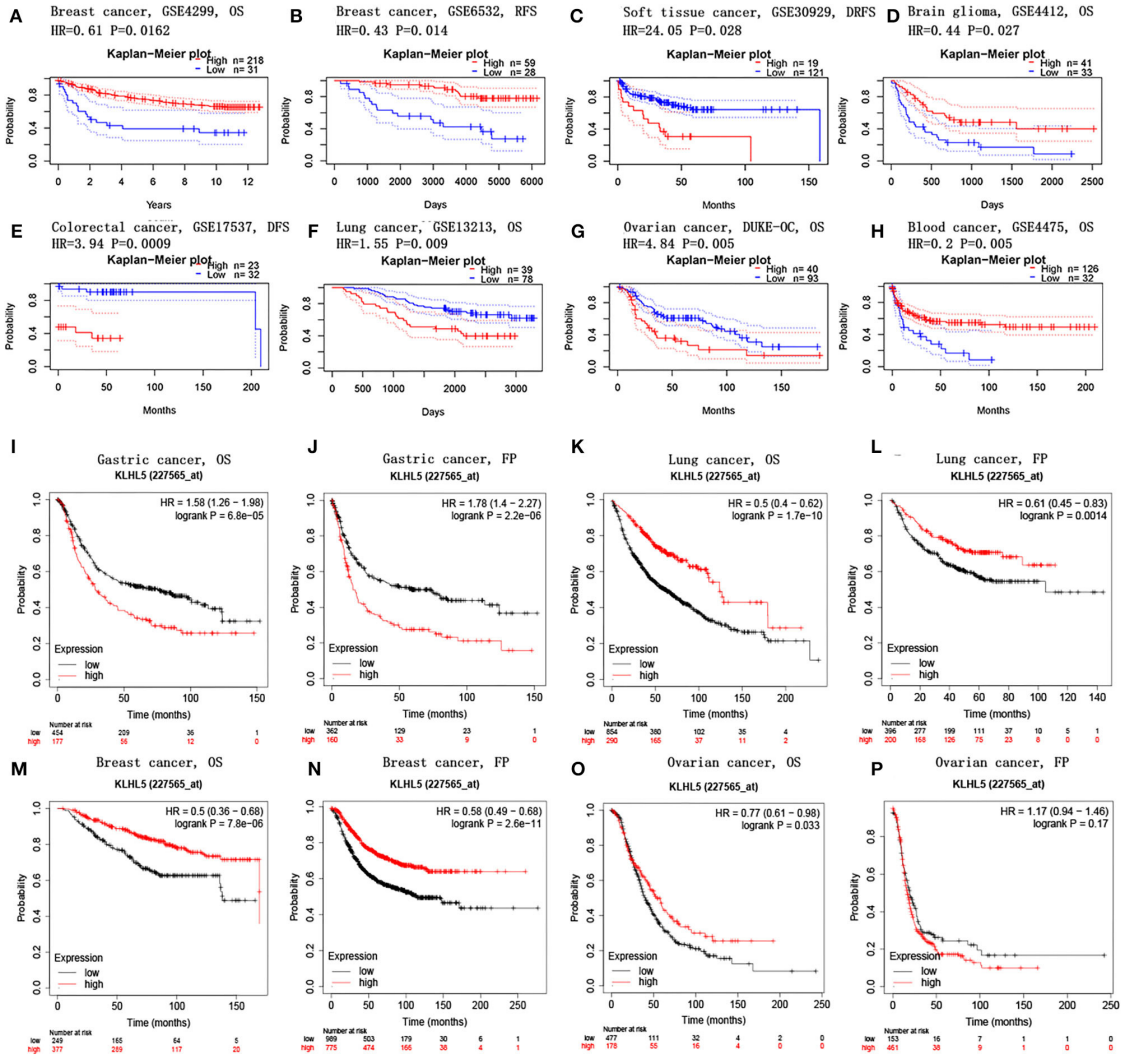
Correlations between KLHL5 and prognosis in different cancers. **(A,B)** Survival curves of DFS and RFS in two breast cancer cohorts. **(C–H)** KLHL5 expression is correlated with DFS in breast cancer **(C)**, OS in brain glioma **(D)**, DFS in colorectal cancer **(E)**, OS in lung cancer **(F)**, OS in ovarian cancer **(G)**, and OS in blood cancer **(H)**. **(I–P)** OS and PFS survival curves of gastric **(I,J)**, lung **(K,L)**, breast **(M,N)**, and ovarian cancer **(O,P)**.

PrognoScan database was explored to investigate the effect of KLHL5 expression on patients' prognosis across 13 tumor types (Supplementary Tables 2–5), and we observed a significant correlation between patient prognostic outcomes and KLHL5 expression in 7 of the 13 types including soft tissue, mammary, brain, colorectal, lung, ovarian, and blood cancer (Figures 2A–H). In addition, to further analyze the correlative significance between KLHL5 expression and prognosis from published cancer microarrays, Kaplan–Meier plotter was employed and it revealed that the overexpression of KLHL5 was significantly associated with a poorer overall survival (OS) (HR 1.58, 95% CI 1.26–1.98, *P* = 6.8e−05) and progression-free survival (PFS) (HR 1.78, 95% CI 1.4–2.27, *P* = 2.2e−06) in gastric cancer patients (Figures 2I, J). Meanwhile, its upregulation was closely related with a better prognosis in patients with lung cancer (OS HR 0.5, 95% CI 0.64–0.62, *P* = 1.7e−10; PFS HR 0.61, 95% CI 0.45–0.83, *P* = 0.0014) and breast cancer (OS HR 0.5, 95% CI 0.36–0.68, *P* = 7.8e−06; PFS HR 0.58, 95% CI 0.49–0.68, *P* = 2.6e−11) (Figures 2K–N). Moreover, the elevated expression of KLHL5 was significantly correlated with a favorable OS (HR 0.77, 95% CI 0.61–0.92, *P* = 0.033) but had no effect on PFS (HR 1.17, 95% CI 0.94–1.46, *P* = 0.17) in ovarian cancer patients (Figures 2O, P).

To better understand the full prospect of the relationship between KLHL5 expression and patient survival in 33 tumor types, we inquired into the GEPIA for survival plots in each cancer type. The results showed that there was a significant correlation between KLHL5 overexpression and a better OS and DFS in KIRC, but a more unfavorable OS and DFS in ACC (adrenocortical carcinoma; Supplementary Figure 1). In addition, overexpression of KLHL5 was correlated with a worse OS in LUAD and MESO, but with a better OS in SKCM (skin cutaneous melanoma). Results from GEPIA and PrognoScan affirmed that KLHL5 was a valuable predictor of prognosis in multiple malignancies.

### KLHL5 Overexpression Is Related With Prognosis in Node-Positive Patients of STAD

As we have discovered that KLHL5 overexpression was associated with worse prognosis in STAD, we further used Kaplan–Meier plotter to examine the association between KLHL5 and patient survival with restricted clinicopathological parameters. We found that expression of KLHL5 was not only significantly correlated with OS and PFS in STAD patients but also in subgroups of different gender, stage, tumor size stage, and node stage; however no such association was found in node-free (OS HR 2.24, *P* = 0.1026; FP HR 2.4, *P* = 0.0748) or M1 group (OS HR 0.68, *P* =0.2454; FP HR 0.79, *P* = 0.443; Table 1). As lymphatic route is the most common way for gastric cancer cell metastasis and lymph node status is directly correlated with patient prognosis (Deng and Liang, 2014), the finding that correlation between KLHL5 and stage N2 has the highest ratio of OS and PFS except for stage 2 and tumor size stage 4 indicates that KLHL5 overexpression has an impact on patient prognosis, possibly by shaping lymph node metastases in gastric cancer.

**Table 1:** The correlation between KLHL5 expression and prognosis in STAD patients with different clinicopathological parameters.

**Clinicopathological characteristics**	**Overall survival (*****N*** **=** **631)**			**Progression-free survival (*****N*** **=** **522)**		
	***N***	**Hazard ratio**	***P*-value**	***N***	**Hazard ratio**	***P*-value**
Sex						
Male	349	1.78 (1.31–2.42)	0.0002	341	1.87 (1.38–2.53)	4.20e−05
Female	187	1.95 (1.27–3)	0.002	179	1.92 (1.26–2.92)	0.0019
Stage						
1	62	0.28 (0.09–0.84)	0.0148	60	0.31 (0.1–0.93)	0.0276
2	135	3.48 (1.36–8.89)	0.0055	131	4.1 (1.46–11.47)	0.0036
3	197	1.65 (1.11–2.47)	0.0132	186	1.86 (1.26–2.75)	0.0016
4	140	1.67 (1.08–2.59)	0.0205	141	1.52 (0.99–2.32)	0.0522
Stage T						
2	241	1.79 (1.27–3.06)	0.002	139	1.95 (1.27–2.99)	0.0017
3	204	1.44 (1.01–2.05)	0.0446	204	1.38 (0.98–1.94)	0.0675
4	38	2.27 (0.97–5.34)	0.0528	39	2.22 (1–4.9)	0.0436
Stage N						
0	74	2.24 (0.83–6.03)	0.1026	72	2.4 (0.89–6.46)	0.0748
1	225	2.02 (1.32–3.08)	0.00095	222	2.17 (1.38–3.42)	0.0006
2	121	1.55 (0.96–2.5)	0.0702	125	1.61 (1.01–2.57)	0.0437
3	76	1.84 (1.08–3.14)	0.023	76	1.96 (1.12–3.44)	0.0167
1+2+3	422	1.91 (1.46–2.5)	1.7e−06	423	1.86 (1.44–2.41)	1.8e−06
Stage M						
0	444	1.9 (1.43–2.53)	6.3e−06	443	1.87 (1.42–2.45)	4.4e−06
1	56	0.68 (0.36–1.3)	0.2454	56	0.79 (0.44–1.43)	0.4436
Lauren classification						
Intestinal	269	1.39 (0.94–2.08)	0.0999	263	1.71 (1.16–2.5)	0.0056
Diffuse	240	1.87 (1.32–2.65)	0.00031	231	1.89 (1.34–2.67)	0.00024
Differentiation						
Poor	121	1.69 (0.94–3.05)	0.0771	121	1.88 (1.07–3.32)	0.0267
Moderate	67	1.71(0.88–3.3)	0.1064	67	1.88 (0.97–3.61)	0.0557

### KLHL5 Is Correlated With Immune Infiltrates in STAD

**Figure 3:**
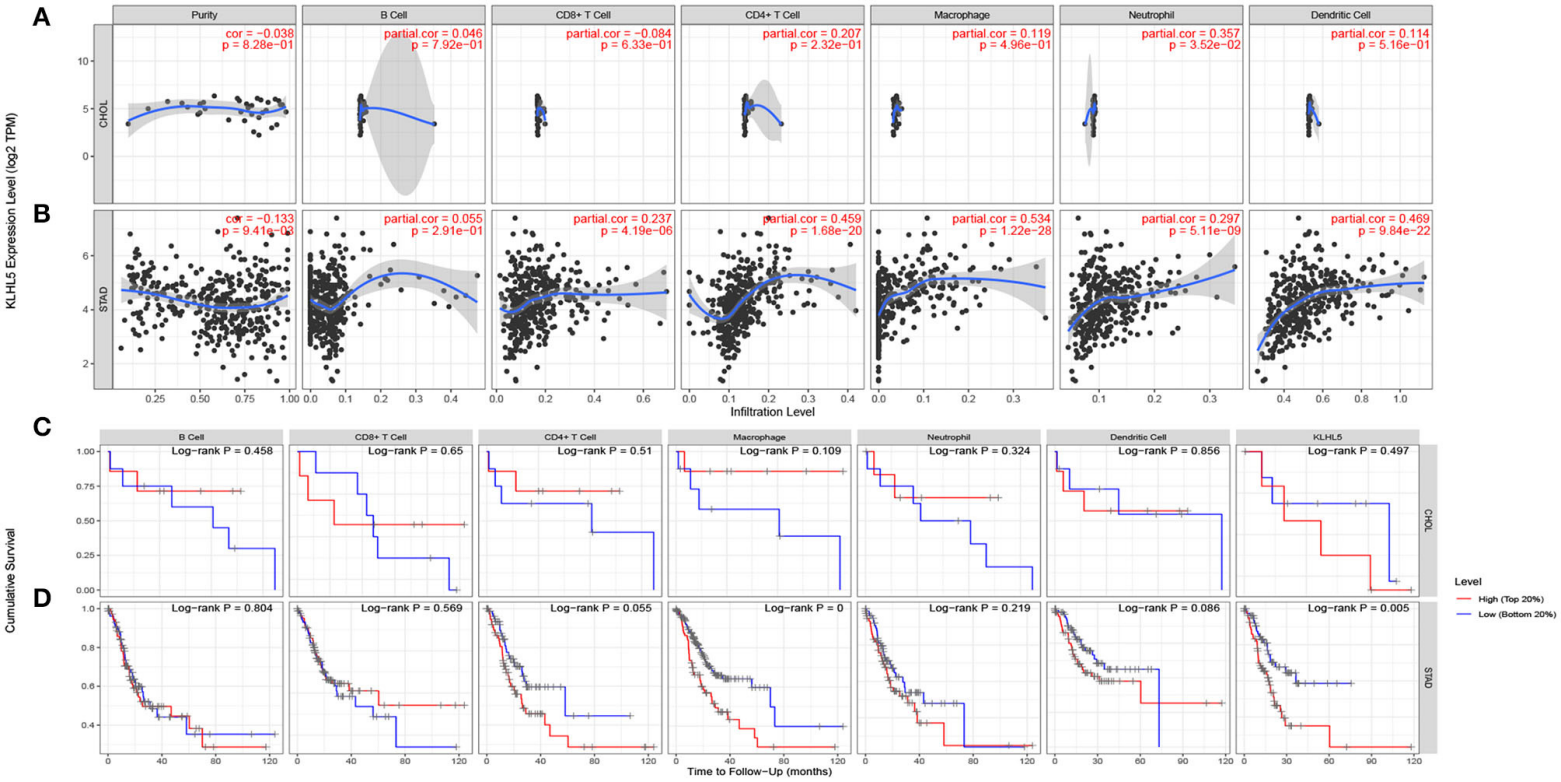
Correlation between KLHL5 expression and inflammatory infiltration in CHOL and STAD. **(A)** KLHL5 is not correlated with tumor purity, B, CD8^+^ T, CD4^+^ T, macrophage, or dendritic cell infiltration in CHOL. It has a weak correlation with the level of neutrophil. **(B)** KLHL5 expression is associated with tumor purity and the infiltration of B, CD8^+^ T, CD4^+^ T, macrophage, neutrophil, and dendritic cells in STAD. **(C)** Kaplan–Meier curves of immune cell infiltration and KLHL5 expression in CHOL. **(D)** Kaplan–Meier curves of immune cell infiltration and KLHL5 expression in SATD.

The inflammatory percolation, representing local anti-tumor immune responses, is identified as an independent factor relevant to sentinel node status and prognosis in patients with malignant melanoma, colorectal, and breast cancer (Ohtani, 2007; Azimi et al., 2012; Ravelli et al., 2017); hence, we explored TIMER database to determine whether the expression of KLHL5 was linked to immunological infiltrates level across 39 types of tumors. After surveying the database, we found there was a significant correlation between KLHL5 with tumor purity in 23 cancers. What is more, KLHL5 expression was associated with the immunocyte level of B cell in 21 types, CD8^+^ T cell in 27 types, CD4^+^ T cell in 25 types, macrophage in 33 types, dendritic cell in 31 types, and neutrophil in 33 cancer types (Supplementary Figures 2A–2AK). The results showed that KLHL5 expression was not correlated with tumor purity (*P* = 8.28e−01), B cell (*P* = 7.92e−01), CD4^+^ T lymphocytes (*P* = 2.32e−01), CD8^+^ T lymphocytes (*P* = 6.33e−01), macrophages (*P* = 4.96e−01), or dendritic cell (*P* = 6.16e−01) level in cholangiocarcinoma (Figure 3A).

Meanwhile, KLHL5 was closely related with level of CD8^+^ T cells (cor = 0.237, *P* = 4.19e−06), CD4^+^ T cells (cor = 0.459, *P* = 4.19e−06), and macrophage (cor = 0.534, *P* = 1.22e−28), neutrophil (cor = 0.297, *P* = 5.1e−09), and dendritic cell (cor = 0.469, *P* = 9.48e−22) infiltration in gastric cancer (Figure 3B). Then Kaplan–Meier curves were plotted to illustrate the correlation between inflammatory infiltration and prognosis and KLHL5 expression, respectively, in CHOL and STAD. The results revealed that immune infiltrate level was not correlated with KLHL5 expression or prognosis in CHOL (Figure 3C). In STAD, although five of the six infiltrates were significantly correlated with KLHL5 expression, only macrophage infiltration and KLHL5 expression were associated with cumulative survival (Figure 3D). This reveals that KLHL5 is an important modulator in gastric cancer immune infiltration, potentially inducing tumor macrophage infiltration.

### KLHL5 Expression Correlates With Particular Immune Markers

**Figure 4:**
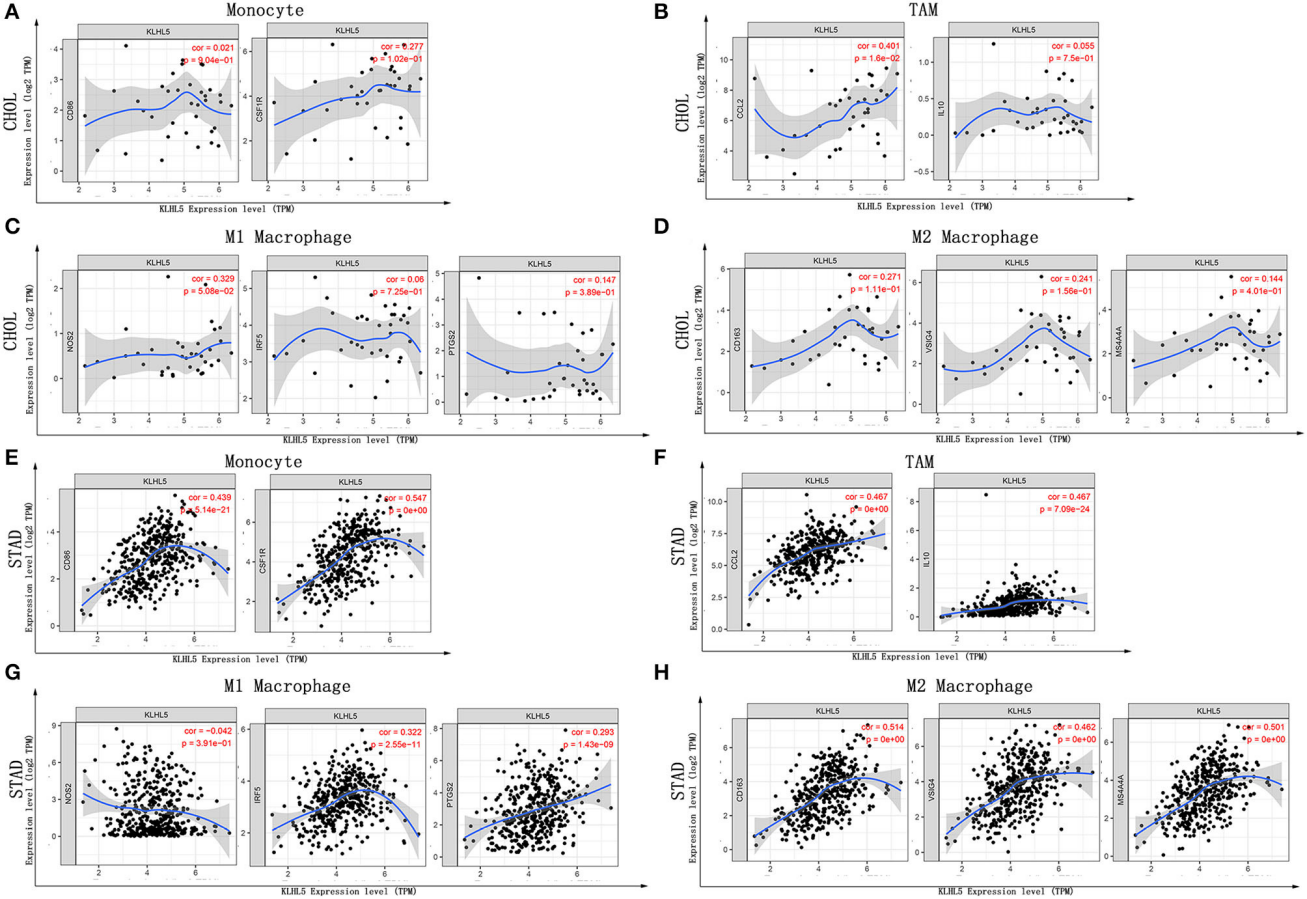
Correlation between KLHL5 and immunocyte marker sets in CHOL and STAD. **(A–D)** The association between KLHL5 and gene markers of monocytes **(A)**, TAMs **(B)**, and M1 **(C)** and M2 macrophages **(D)** in CHOL. **(E–H)** Scatterplots of correlation between KLHL5 and gene markers of monocytes **(E)**, TAMs **(F)**, and M1 **(G)** and M2 macrophages **(H)** in STAD.

Next, to further substantiate the relation between KLHL5 and immunocyte level, TIMER site and GEPIA were surveyed to examine the correlation between immunocyte gene makers and KLHL5 expression in gastric adenocarcinoma, with cholangiocarcinoma serving as the control. The correlation between KLHL5 and gene markers associated with immunocyte subsets was extracted and results were adjusted according to the tumor purity. The results showed that KLHL5 was significantly correlated with majority (48/57) of the gene markers in STAD (Table 2); however, only 4 of the 57 markers were found to be related with KLHL5 expression in CHOL. In STAD, markers of monocyte (CD86, CSF1R), TAM (CCL2, IL10), M1 macrophage (IRF5, PTGS2), and M2 macrophage (CD163, VSIG4, MS4A4A) were closely correlated with KLHL5 (*P* < 0.0001; Figures 4A–H), which was also corroborated by GEPIA (Table 3) later. This indicated that KLHL5 may be involved in the macrophage polarization in STAD. KLHL5 overexpression in gastric cancer was correlated with enhanced dendritic cell infiltration in TIMER; on par with this, the expression of dendritic cell markers was also related with KLHL5 level in GEPIA (Table 3). This hinted that KLHL5 is a key factor in tumor dendritic cell penetration. Dendritic cells are capable of stimulating tumor metastasis by boosting Tregs responses and suppressing CD8^+^ T-cell cytotoxic ability (Facciabene et al., 2012; Wang et al., 2017). More studies are needed to confirm KLHL5 function in dendritic cell regulation and tumor metastasis in the future. Moreover, KLHL5 was found to be significantly associated with Tregs and exhausted T-cell biomarkers (Table 2), suggesting that KLHL5 may have an impact on escape of immune surveillance in gastric cancer, which needs further research to be expounded.

**Table 2:** Correlation between KLHL5 and gene markers of immune infiltrates from TIMER database.

**Description**	**Gene markers**	**STAD**				**CHOL**			
		**None**		**Purity**		**None**		**Purity**	
		**Cor**	***P***	**Cor**	***P***	**Cor**	***P***	**Cor**	***P***
CD8^+^ T cell	CD8A	0.336	^***^	0.332	^***^	−0.066	0.702	−0.092	0.597
	CD8B	0.321	^***^	0.329	^***^	−0.394	0.018	−0.434	^*^
T cell (general)	CD3D	0.301	^***^	0.291	^***^	−0.136	0.429	−0.181	0.298
	CD3E	0.314	^***^	0.306	^***^	−0.032	0.825	−0.067	0.701
	CD2	0.359	^***^	0.353	^***^	−0.006	0.727	−0.098	0.575
B cell	CD19	0.357	^***^	0.342	^***^	0.032	0.854	0.0150	0.932
	CD79A	0.309	^***^	0.285	^***^	−0.023	0.896	−0.046	0.291
Monocyte	CD86	0.439	^***^	0.443	^***^	0.021	0.904	0.001	0.996
	CD115 (CSF1R)	0.574	^***^	0.547	^***^	0.277	0.102	0.287	0.095
TAM	CCL2	0.467	^***^	0.465	^***^	0.401	0.016	0.404	0.016
	CD68	0.235	^***^	0.226	^***^	−0.005	0.977	−0.016	0.925
	IL10	0.467	^***^	0.473	^***^	0.055	0.750	0.040	0.819
M1 macrophage	INOS (NOS2)	−0.042	0.390	0.048	0.350	0.329	0.051	0.329	0.053
	IRF5	0.322	^***^	0.326	^***^	0.060	0.725	0.053	0.762
	COX2(PTGS2)	0.293	^***^	0.282	^***^	0.147	0.389	0.144	0.409
M2 macrophage	CD163	0.514	^***^	0.515	^***^	0.271	0.111	0.290	0.091
	VSIG4	0.462	^***^	0.479	^***^	0.241	0.156	0.251	0.146
	MS4A4A	0.501	^***^	0.510	^***^	0.144	0.401	0.154	0.378
Neutrophils	CD66b(CEACAM8)	0.053	0.281	0.081	0.116	0.047	0.748	0.047	0.791
	CD11b(ITGAM)	0.524	^***^	0.533	^***^	0.531	^*^	0.539	^**^
	CCR7	0.459	^***^	0.457	^***^	0.049	0.774	0.034	0.846
Natural killer cell	KIR2DL1	0.191	^***^	0.195	^**^	−0.225	0.186	−0.234	0.177
	KIR2DL3	0.109	0.025	0.092	0.073	0.079	0.648	0.075	0.669
	KIR2DL4	0.015	0.758	−0.038	0.466	−0.335	0.045	−0.353	0.037
	KIR3DL1	0.155	^*^	0.135	^*^	−0.335	0.033	−0.364	0.031
	KIR3DL2	0.161	^**^	0.149	^*^	−0.042	0.808	−0.043	0.806
	KIR3DL3	0.060	0.159	−0.054	0.291	−0.004	0.979	−0.001	0.956
	KIR2DS4	0.104	0.033	0.092	0.075	−0.093	0.589	−0.010	0.567
Dendritic cell	HLA–DPB1	0.290	^***^	0.277	^***^	−0.125	0.375	0.189	0.276
	HLA-DQB1	0.156	^*^	0.143	^*^	−0.120	0.484	0.114	0.513
	HLA-DRA	0.225	^***^	0.213	^***^	−0.093	0.589	−0.128	0.465
	HLA-DPA1	0.247	^***^	0.230	^***^	−0.083	0.629	−0.116	0.508
	BDCA-1(CD1C)	0.445	^***^	0.443	^***^	0.142	0.410	0.14	0.424
	BDCA-4(NRP1)	0.669	^***^	0.673	^***^	0.511	^*^	0.529	^*^
	CD11c(ITGAX)	0.467	^***^	0.458	^***^	0.201	0.238	0.212	0.222
Th1	T-bet(TBX 21)	0.338	^***^	0.343	^***^	−0.152	0.376	−0.218	0.208
	STAT4	0.497	^***^	0.500	^***^	0.164	0.339	0.16	0.359
	STAT1	0.229	^***^	0.223	^***^	0.396	0.017	0.396	0.019
	IFN-y(IFNG)	0.095	0.053	0.083	0.011	−0.174	0.311	−0.217	0.211
	TNF-a(TNF)	0.217	^***^	0.191	^**^	0.257	0.130	0.254	0.140
Th2	GATA3	0.413	^***^	0.429	^***^	−0.048	0.780	−0.083	0.634
	STAT6	0.278	^***^	0.278	^***^	0.423	0.011	0.425	0.011
	STAT5A	0.462	^***^	0.473	^***^	0.335	0.047	0.334	0.049
	IL13	0.145	*	0.161	*	0.033	0.849	0.024	0.890
Tfh	BCL6	0.601	^***^	0.591	^***^	0.117	0.500	0.114	0.515
	IL21	0.177	^**^	0.169	^**^	0.089	0.605	0.082	0.640
Th17	STAT3	0.538	^***^	0.535	^***^	0.435	*	0.435	*
	IL17A	−0.050	0.312	−0.066	0.202	−0.196	0.253	−0.211	0.224
Treg	FOXP3	0.349	^***^	0.337	^***^	0.075	0.664	0.065	0.713
	CCR8	0.463	^***^	0.464	^***^	0.113	0.510	0.107	0.539
	STAT5B	0.645	^***^	0.639	^***^	0.380	0.023	0.379	0.025
	TGFB(TGFB1)	0.558	^***^	0.558	^***^	0.510	*	0.522	*
T-cell exhaustion	PD-1(PDCD1)	0.297	^***^	0.299	^***^	0.235	0.167	0.234	0.176
	CTLA4	0.322	^***^	0.316	^***^	−0.101	0.558	−0.120	0.493
	LAG3	0.223	^***^	0.210	^***^	−0.107	0.535	−0.130	0.456
	TIM-3(HAVCR2)	0.405	^***^	0.409	^***^	0.175	0.306	178	0.306
	GZMB	0.082	0.095	0.046	0.037	−0.160	0.350	−0.197	0.257

*STAD, stomach adenocarcinoma; CHOL, cholangiocarcinoma; TAM, tumor-associated macrophage; Th, T helper cell; Tfh, follicular helper T cell; Treg, regulatory T cell; Cor, R-value of Spearman's correlation; None, correlation without adjustment; Purity, correlation adjusted by tumor purity*.

* *P < 0.01*;

** *P < 0.001*;

*** *P < 0.0001*.

**Table 3:** Correlation between KLHL5 expression and gene markers of monocyte, M1 macrophage, and M2 macrophage.

		**STAD**			
		**Tumor**		**Normal**	
**Description**	**Gene markers**	**R**	***P***	**R**	***P***
Monocyte	CD86	0.43	^***^	−0.27	0.11
	CD115(CSF1R)	0.53	^***^	−0.09	0.6
TAM	CCL2	0.46	^***^	−0.51	0.017
	CD68	0.22	^***^	−0.62	^***^
	IL10	0.46	^***^	−0.085	0.62
M1 macrophage	INOS(NOS2)	−0.078	.45	−0.16	0.35
	IRF5	0.34	^***^	−0.23	0.18
	CPX2(PTGS2)	0.32	^***^	0.63	^***^
M2 macrophage	CD163	0.46	^***^	0.49	^*^
	VSIG4	0.44	^***^	0.2	0.24
	MS4A4A	0.48	^***^	0.39	0.02

*STAD, stomach adenocarcinoma; R, value of Spearman's correlation*.

* *P < 0.01*;

** *P < 0.001*;

*** *P < 0.0001*.

## Discussion

KLHL5 is a widely expressed protein encoding gene in benign and malignant tissues. Although its full functions remain uncertain, it is clear that KLHL5 knockdown inhibits cell proliferation in certain cancer cell lines (Schleifer et al., 2018). Here, we report KLHL5 expression correlates with patient clinical outcomes in multiple cancer types. High level of KLHL5 in STAD is related with a poorer OS and DFS; particularly, it is significantly correlated with prognosis in lymph node–positive patients, which indicates that KLHL5 is a potential biomarker of tumor metastasis. Moreover, our study has revealed a strong correlation between KLHL5 and infiltrate level and different immune-related gene sets. Therefore, our study provides insight into KLHL5 as a potential prognostic related marker in STAD.

**Figure 5:**
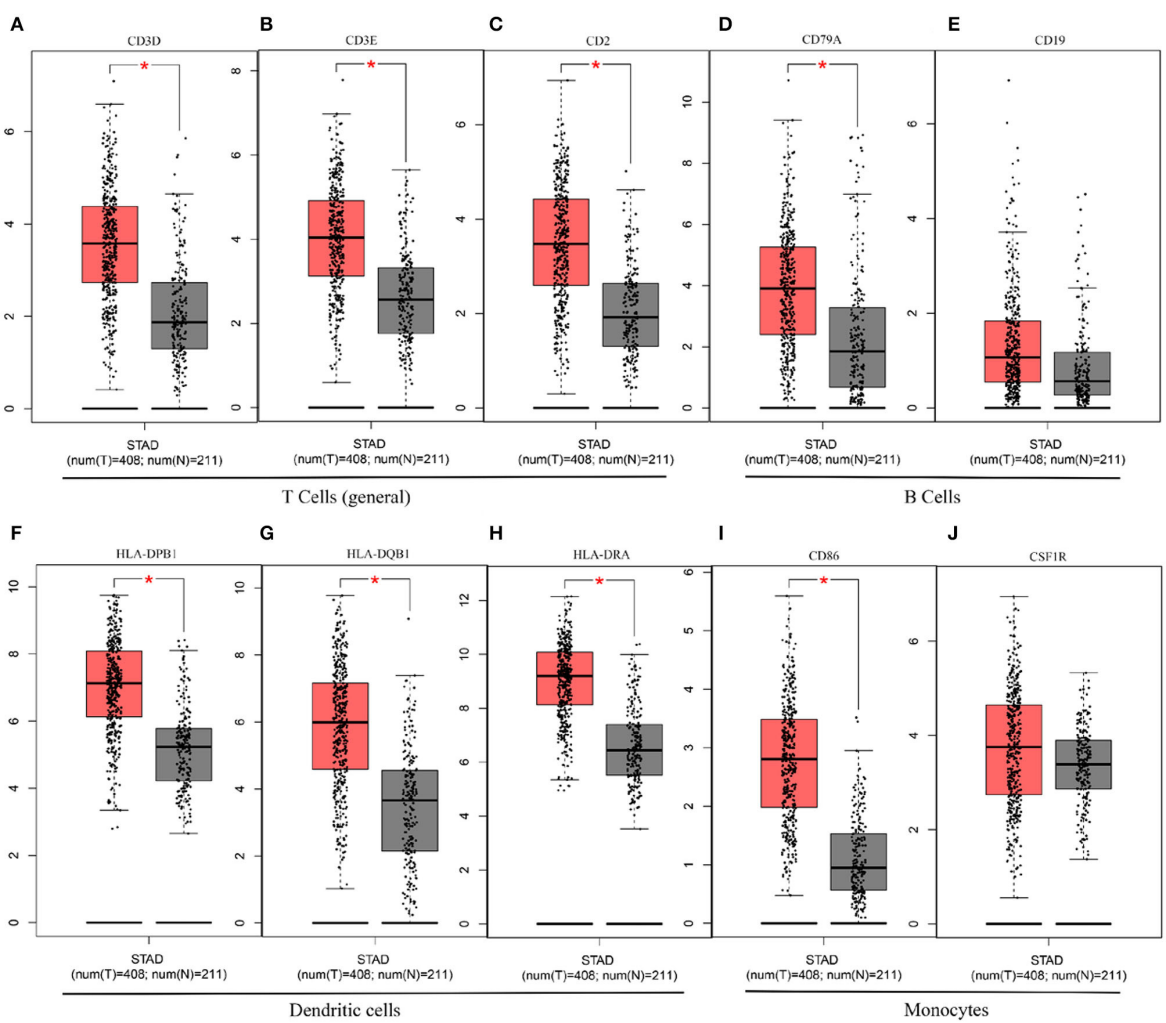
The differential expression of CD3D **(A)**, CD3E **(B)**, CD2 **(C)**, CD79A **(D)**, CD19 **(E)**, HLA-DPB1 **(F)**, HLA-DQB1 **(G)**, HLA-DRA **(H)**, CD86 **(I)**, and CSF1R **(J)** in gastric cancer.

In this study, we mined Oncomine and GEPIA database for the landscape of KLHL5 in 33 types of cancers, and observed varied expression of KLHL5 between tumor tissues and benign controls in cancers. Oncomine data showed that the overexpression of KLHL5 was identified in many malignancies like brain, cervical, breast, esophageal, colorectal, and gastric tumors, whereas it was downregulated in cancer tissues of bladder, lung, prostate, and so on in some microarrays (Figure 1A). TCGA data revealed that the level of KLHL5 expression was relatively higher in CHOL, ESCA, HNSC, LIHC, LUAD, LUSC, and STAD in comparison with benign tissues. In contrast, its expression was significantly lower in READ, KICH, KIRC, KIRP, BRCA, PRAD, BLCA, and UCEC compared with adjacent benign samples (Figure 1B). The variant expression level of KLHL5 in the same cancer from different databases or even the same databases may be attributed to different data collection approaches and diversified molecular functions. Moreover, results from GEPIA showed no significant correlation between KLHL5 expression and patient OS or DFS in STAD, which was not concordant with that from Kaplan–Meier plotter database. This may be attributed to different sample volumes (631 in Kaplan–Meier Plotter and 384 in GEPIA) included in this analysis (Figures 2I,J; Supplementary Figures 1BA,BB). Across the TCGA database, the expression of KLHL5 correlates with prognosis in mammary, brain, soft tissue, colorectal, lung, ovarian, and blood cancer (Figures 2A–H). Also, survival analysis of Kaplan–Meier plotter database showed that the KLHL5 overexpression foreboded disappointing OS and PFS in gastric neoplasm patients (Figures 2I,J). Meanwhile, its upregulation was correlated with a relatively favorable survival in lung cancer and breast cancer patients (Figures 2K–N). Furthermore, KLHL5 expression correlated significantly with OS and PFS in subgroups divided by clinicopathological features, such as gender, stage, tumor stage, and node stage excluding node free or distant metastasis. Also, the correlation between KLHL5 and stage N2 has the highest ratio of OS and PFS except for stage 2 and tumor size stage 4 (Table 1). All these results indicate that KLHL5 is a reliable prognostic biomarker in STAD. Another finding in our study is that the level of inflammatory infiltrates is correlative of KLHL5 in different types of cancers, especially in STAD. Our analyses displayed a moderate positive relationship between KLHL5 and infiltration degree of CD4^+^ T, macrophage, and dendritic cells. What is more, a weak positive correlation was also detected between KLHL5 and infiltrating level of CD8^+^ T cells and neutrophil (Figures 3A,B). Furthermore, a strong correlation between KLHL5 and immune-related gene sets was found (Table 2), indicating that KLHL5 has a great impact on immune infiltration in STAD. We analyzed the expression profiles of immune cell markers in GEPIA and the results showed makers of T cells (CD3D, CD3E, and CD2) (Figures 5A–C), B cells (CD79A and CD19) (Figures 5D,E), dendritic cells (HLA-DPB1, HLA-DQB1, and HLA-DRA) (Figures 5F–H), and monocytes (CD86 and CSF1R) (Figures 5I,J) are overexpressed in gastric cancer samples compared with normal controls. This suggests the relatively high immune infiltration status in gastric cancer.

We observed that there was a weak to moderate correlation between KLHL5 and gene markers of all three macrophage subtypes including TAMs, M1, and M2 macrophages (Table 3). This implicates that KLHL5 can regulate TAM polarization. Another finding was that KLHL5 expression had a weak to strong correlation with markers of Tregs and exhausted T cell (Table 2), indicating that KLHL5 was capable of promoting Tregs reaction to block T cell–mediated cytotoxicity. Moreover, KLHL5 was closely related with other T-cell markers including different subtypes of T-helper cells in STAD. This hints that KLHL5 may modulate T lymphocyte immunity in gastric cancer. These results suggest that KLHL5 plays extensive roles in cancer inflammatory infiltration.

In summary, elevated KLHL5 expression is correlated with worse prognosis and higher immunological infiltration in multiple malignancies, particularly in STAD. KLHL5 is potentially involved in TAM polarization, Treg responses, and T-cell responses. Thus, KLHL5 is identified as a reliable predictor of prognosis in gastric cancer patients.

## Data Availability Statement

The original contributions presented in the study are included in the article/Supplementary Materials, further inquiries can be directed to the corresponding author.

## Ethics Statement

Ethical review and approval was not required for the study on human participants in accordance with the local legislation and institutional requirements. Written informed consent for participation was not required for this study in accordance with the national legislation and the institutional requirements. Ethical review and approval was not required for the animal study because this study was conducted with bioinformatic tools, all the data were extracted from public databases.

## Author Contributions

SL conceived the project and reviewed the article. QW and GY participated in data analysis and wrote the article. JL, JT, and AL participated in discussion. All authors contributed to the article and approved the submitted version.

## Conflict of Interest

The authors declare that the research was conducted in the absence of any commercial or financial relationships that could be construed as a potential conflict of interest.

## References

[B1] Adams J. Kelso R. Cooley L. (2000). The kelch repeat superfamily of proteins: propellers of cell function. Trends Cell Biol. 10, 17–24. 10.1016/S0962-8924(99)01673-6 10603472

[B2] Angers S. Thorpe C. J. Biechele T. L. Goldenberg S. J. Zheng N. MacCoss M. J.. (2006). The KLHL12-Cullin-3 ubiquitin ligase negatively regulates the Wnt-beta-catenin pathway by targeting Dishevelled for degradation. Nat. Cell Biol. 8, 348–357. 10.1038/ncb1381 16547521

[B3] Azimi F. Scolyer R. A. Rumcheva M. M. Murali R. McCarthy S. W.. (2012). Tumor-infiltrating lymphocyte grade is an independent predictor of sentinel lymph node status and survival in patients with cutaneous melanoma. J. Clin. Oncol. 30, 2678–2683. 10.1200/JCO.2011.37.8539 22711850

[B4] Bizzaro N. Antico A. Villalta D. (2018). Autoimmunity and gastric cancer. Int. J. Mol. Sci. 19:377. 10.3390/ijms19020377 29373557 PMC5855599

[B5] Deng J. Y. Liang H. (2014). Clinical significance of lymph node metastasis in gastric cancer. World J. Gastroenterol. 20, 3967–3975. 10.3748/wjg.v20.i14.3967 24744586 PMC3983452

[B6] Dhanoa B. S. Cogliati T. Satish A. G. Bruford E. A. Friedman J. S. (2013). Update on the Kelch-like (KLHL) gene family. Hum. Genomics 7:13. 10.1186/1479-7364-7-13 23676014 PMC3658946

[B7] Facciabene A. Motz G. T. Coukos G. (2012). T-regulatory cells: key players in tumor immune escape and angiogenesis. Cancer Res. 72, 2162–2171. 10.1158/0008-5472.CAN-11-3687 22549946 PMC3342842

[B8] Itoh K. Wakabayashi N. Katoh Y. Ishii T. Igarashi K. Engel J. D.. (1999). Keap1 represses nuclear activation of antioxidant responsive elements by Nrf2 through binding to the amino-terminal Neh2 domain. Genes Dev. 13, 76–86. 10.1101/gad.13.1.76 9887101 PMC316370

[B9] Kim S. C. Hong C. W. Jang S. G. Kim Y. A. Yoo B. C. Shin Y. K.. (2018). Establishment and characterization of paired primary and peritoneal seeding human colorectal cancer cell lines: identification of genes that mediate metastatic potential. Transl. Oncol. 11, 1232–1243. 10.1016/j.tranon.2018.07.014 30114595 PMC6097464

[B10] Kroll J. Shi X. Caprioli A. Liu H. H. Waskow C. Lin K. M.. (2005). The BTB-kelch protein KLHL6 is involved in B-lymphocyte antigen receptor signaling and germinal center formation. Mol. Cell. Biol. 25, 8531–8540. 10.1128/MCB.25.19.8531-8540.2005 16166635 PMC1265761

[B11] Lánczky A. Nagy Á. Bottai G. Munkácsy G. Szabó A. Santarpia L.. (2016). miRpower: a web-tool to validate survival-associated miRNAs utilizing expression data from 2178 breast cancer patients. Breast Cancer Res. Treat 160, 439–446. 10.1007/s10549-016-4013-7 27744485

[B12] Le D. T. Uram J. N. Wang H. Bartlett B. R. Kemberling H. Eyring A. D.. (2015). PD-1 blockade in tumors with mismatch-repair deficiency. N. Engl. J. Med. 372, 2509–2520. 10.1056/NEJMoa1500596 26028255 PMC4481136

[B13] Li T. Fan J. Wang B. Traugh N. Chen Q. Liu J. S.. (2017). TIMER: a web server for comprehensive analysis of tumor-infiltrating immune cells. Cancer Res. 77, e108–e110. 10.1158/0008-5472.CAN-17-0307 29092952 PMC6042652

[B14] Liu X. R. Wang W. Li H. M. (2020). KLHL22 promotes malignant melanoma growth in vitro and in vivo by activating the PI3K/Akt/mTOR signaling pathway. Neoplasma 67, 1106–1113. 10.4149/neo_2020_190923N954 32484697

[B15] Mei Z. Z. Chen X. Y. Hu S. W. Wang N. Ou X. L. Wang J.. (2016). Kelch-like protein 21 (KLHL21) targets IκB kinase-β to regulate nuclear factor κ-light chain enhancer of activated B cells (NF-κB) signaling negatively. J. Biol. Chem. 291, 18176–18189. 10.1074/jbc.M116.715854 27387502 PMC5000066

[B16] Metzger T. Kleiss C. Sumara I. (2013). CUL3 and protein kinases: insights from PLK1/KLHL22 interaction. Cell Cycle 12, 2291–2296. 10.4161/cc.25369 24067371 PMC3755079

[B17] Mizuno H. Kitada K. Nakai K. Sarai A. (2009). PrognoScan: a new database for meta-analysis of the prognostic value of genes. BMC Med. Genomics 2:18. 10.1186/1755-8794-2-18 19393097 PMC2689870

[B18] Moehler M. Delic M. Goepfert K. Aust D. Grabsch H. I. Halama N.. (2016). Immunotherapy in gastrointestinal cancer: recent results, current studies and future perspectives. Eur. J. Cancer 59, 160–170. 10.1016/j.ejca.2016.02.020 27039171

[B19] Murata M. (2018). Inflammation and cancer. Environ. Health Prev. Med. 23, 50. 10.1186/s12199-018-0740-1 30340457 PMC6195709

[B20] Muro K. Chung H. C. Shankaran V. Geva R. Catenacci D. Gupta S.. (2016). Pembrolizumab for patients with PD-L1-positive advanced gastric cancer (KEYNOTE-012): a multicentre, open-label, phase 1b trial. Lancet Oncol. 17, 717–726. 10.1016/S1470-2045(16)00175-3 27157491

[B21] Ohtani H. (2007). Focus on TILs: prognostic significance of tumor infiltrating lymphocytes in human colorectal cancer. Cancer Immun. 7:4. 17311363 PMC2935759

[B22] Osmani L. Askin F. Gabrielson E. Li Q. K. (2018). Current WHO guidelines and the critical role of immunohistochemical markers in the subclassification of non-small cell lung carcinoma (NSCLC): moving from targeted therapy to immunotherapy. Semin. Cancer Biol. 52(Pt. 1), 103–109. 10.1016/j.semcancer.2017.11.019 29183778 PMC5970946

[B23] Overman M. J. McDermott R. Leach J. L. Lonardi S. Lenz H. J. Morse M. A.. (2017). Nivolumab in patients with metastatic DNA mismatch repair-deficient or microsatellite instability-high colorectal cancer (CheckMate 142): an open-label, multicentre, phase 2 study. Lancet Oncol. 18, 1182–1191. 10.1016/S1470-2045(17)30422-9 28734759 PMC6207072

[B24] Perez-Torrado R. Yamada D. Defossez P. A. (2006). Born to bind: the BTB protein-protein interaction domain. Bioessays 28, 1194–1202. 10.1002/bies.20500 17120193

[B25] Powell J. A. Pitman M. R. Zebol J. R. Moretti P. A. B. Neubauer H. A. Davies L. T.. (2019). Kelch-like protein 5-mediated ubiquitination of lysine 183 promotes proteasomal degradation of sphingosine kinase 1. Biochem. J. 476, 3211–3226. 10.1042/BCJ20190245 31652307

[B26] Procaccio L. Schirripa M. Fassan M. Vecchione L. Bergamo F. Prete A. A.. (2017). Immunotherapy in gastrointestinal cancers. Biomed Res. Int. 2017:4346576. 10.1155/2017/4346576 28758114 PMC5512095

[B27] Ralph C. Elkord E. Burt D. J. O'Dwyer J. F. Austin E. B. Stern P. L.. (2010). Modulation of lymphocyte regulation for cancer therapy: a phase II trial of tremelimumab in advanced gastric and esophageal adenocarcinoma. Clin. Cancer Res. 16, 1662–1672. 10.1158/1078-0432.CCR-09-2870 20179239

[B28] Ravelli A. Roviello G. Cretella D. Cavazzoni A. Biondi A. Cappelletti M. R.. (2017). Tumor-infiltrating lymphocytes and breast cancer: beyond the prognostic and predictive utility. Tumour Biol. 39:1010428317695023. 10.1177/1010428317695023 28378631

[B29] Rhodes D. R. Kalyana-Sundaram S. Mahavisno V. Varambally R. Yu J. Briggs B. B.. (2007). Oncomine 3.0: genes, pathways, and networks in a collection of 18,000 cancer gene expression profiles. Neoplasia 9, 166–180. 10.1593/neo.07112 17356713 PMC1813932

[B30] Schleifer R. J. Li S. Nechtman W. Miller E. Bai S. Sharma A.. (2018). KLHL5 knockdown increases cellular sensitivity to anticancer drugs. Oncotarget 9, 37429–37438. 10.18632/oncotarget.26462 30647843 PMC6324770

[B31] Siegel R. L. Miller K. D. Jemal A. (2017). Cancer Statistics, 2017. CA Cancer J. Clin. 67, 7–30. 10.3322/caac.21387 28055103

[B32] Tang Z. Li C. Kang B. Gao G. Li C. Zhang Z. (2017). GEPIA: a web server for cancer and normal gene expression profiling and interactive analyses. Nucleic Acids Res. 45, W98–W102. 10.1093/nar/gkx247 28407145 PMC5570223

[B33] Wang F. Meng W. Wang B. Qiao L. (2014). Helicobacter pylori-induced gastric inflammation and gastric cancer. Cancer Lett. 345, 196–202. 10.1016/j.canlet.2013.08.016 23981572

[B34] Wang H. Franco F. Ho P. C. (2017). Metabolic regulation of tregs in cancer: opportunities for immunotherapy. Trends Cancer 3, 583–592. 10.1016/j.trecan.2017.06.005 28780935

[B35] Wang S. Zhou Z. Ying K. Tang R. Huang Y. Wu C.. (2001). Cloning and characterization of KLHL5, a novel human gene encoding a kelch-related protein with a BTB domain. Biochem. Genet. 39, 227–238. 10.1023/A:1010203114697 11590829

[B36] Waniczek D. Lorenc Z. Snietura M. Wesecki M Kopec A. Muc-Wierzgoń M. (2017). Tumor-associated macrophages and regulatory t cells infiltration and the clinical outcome in colorectal cancer. Arch. Immunol. Ther. Exp. 65, 445–454. 10.1007/s00005-017-0463-9 28343267 PMC5602054

[B37] Zhang H. Liu H. Shen Z. Lin C. Wang X. Qin J.. (2018). Tumor-infiltrating neutrophils is prognostic and predictive for postoperative adjuvant chemotherapy benefit in patients with gastric cancer. Ann. Surg. 267, 311–318. 10.1097/SLA.0000000000002058 27763900

[B38] Zhao B. Payne W. G. Sai J. Lu Z. Olejniczak E. T. Fesik S. W. (2020). Structural elucidation of peptide binding to KLHL-12, a substrate specific adapter protein in a Cul3-ring E3 ligase complex. Biochemistry 59, 964–969. 10.1021/acs.biochem.9b01073 32032490

