# Supplementary figures and images for: KLHL5 Is a Prognostic-Related Biomarker and Correlated With Immune Infiltrates in Gastric Cancer
Source: Front Mol Biosci. 2020 Dec 10;7:599110. doi: 10.3389/fmolb.2020.599110 (PMC7758449; doi:10.3389/fmolb.2020.599110)

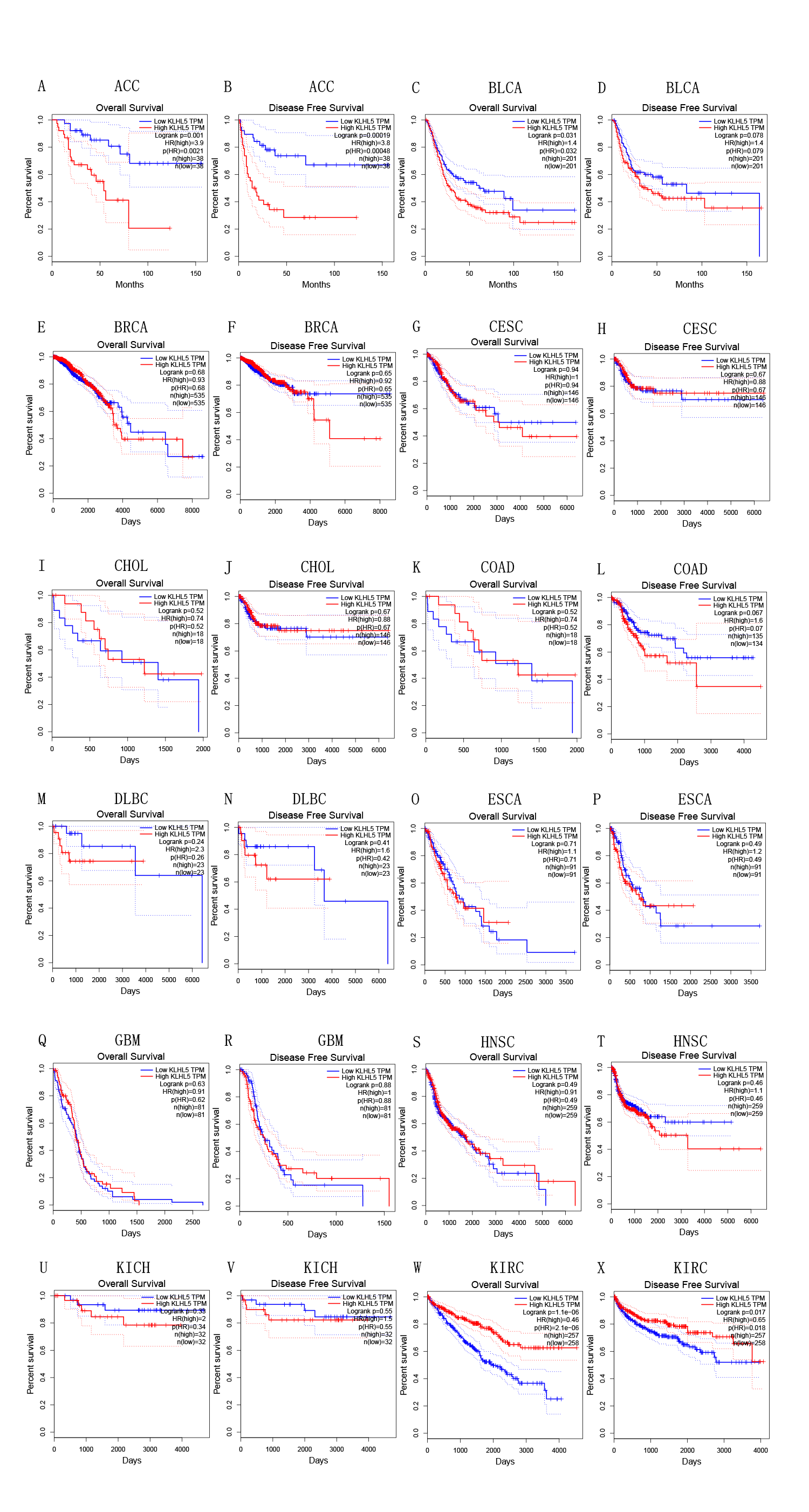

Supplement: Supplementary file 1 [file Image_1.TIF]

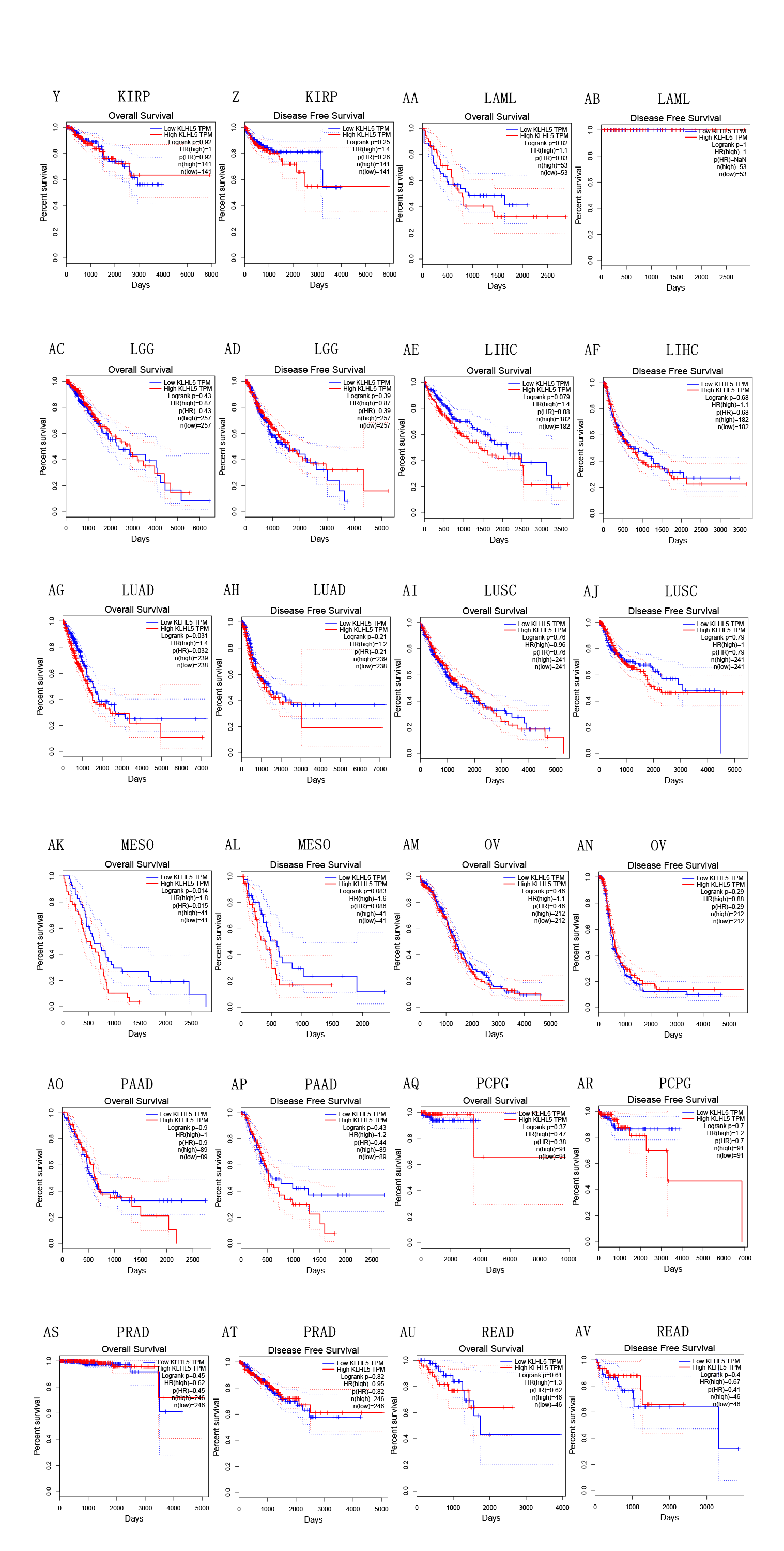

Supplement: Supplementary file 2 [file Image_2.TIF]

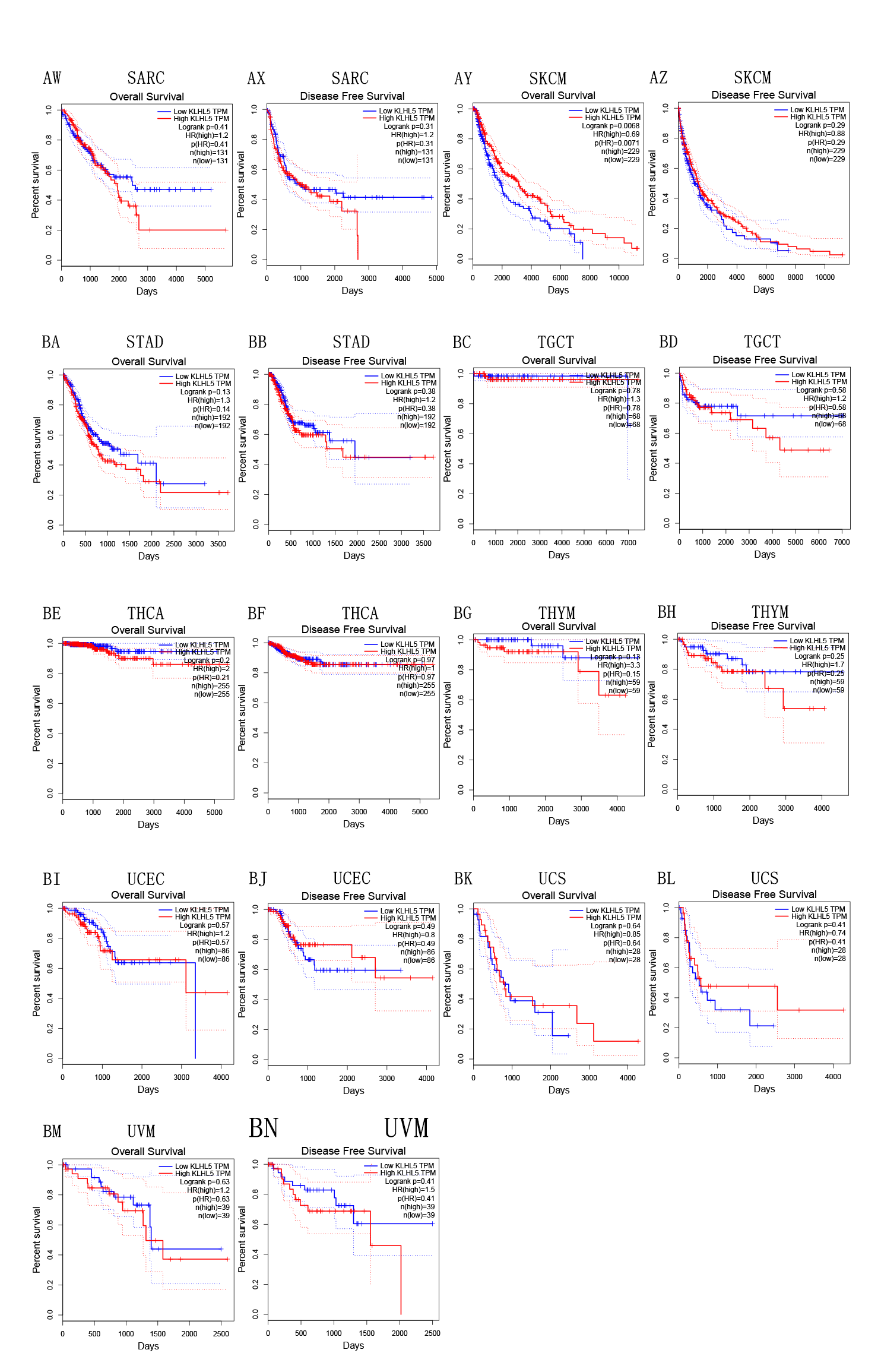

Supplement: Supplementary file 3 [file Image_3.TIF]

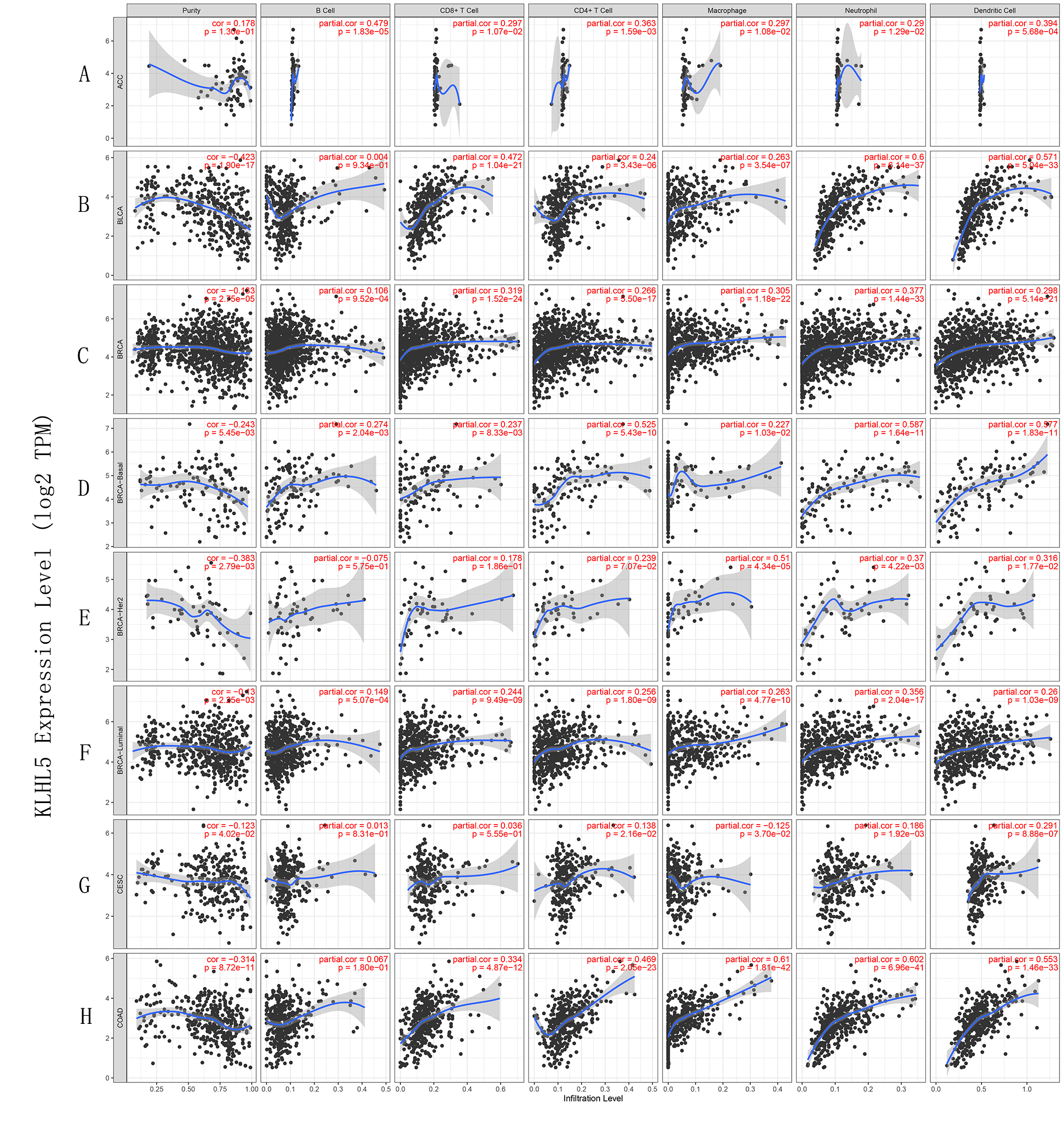

Supplement: Supplementary file 4 [file Image_4.JPEG]

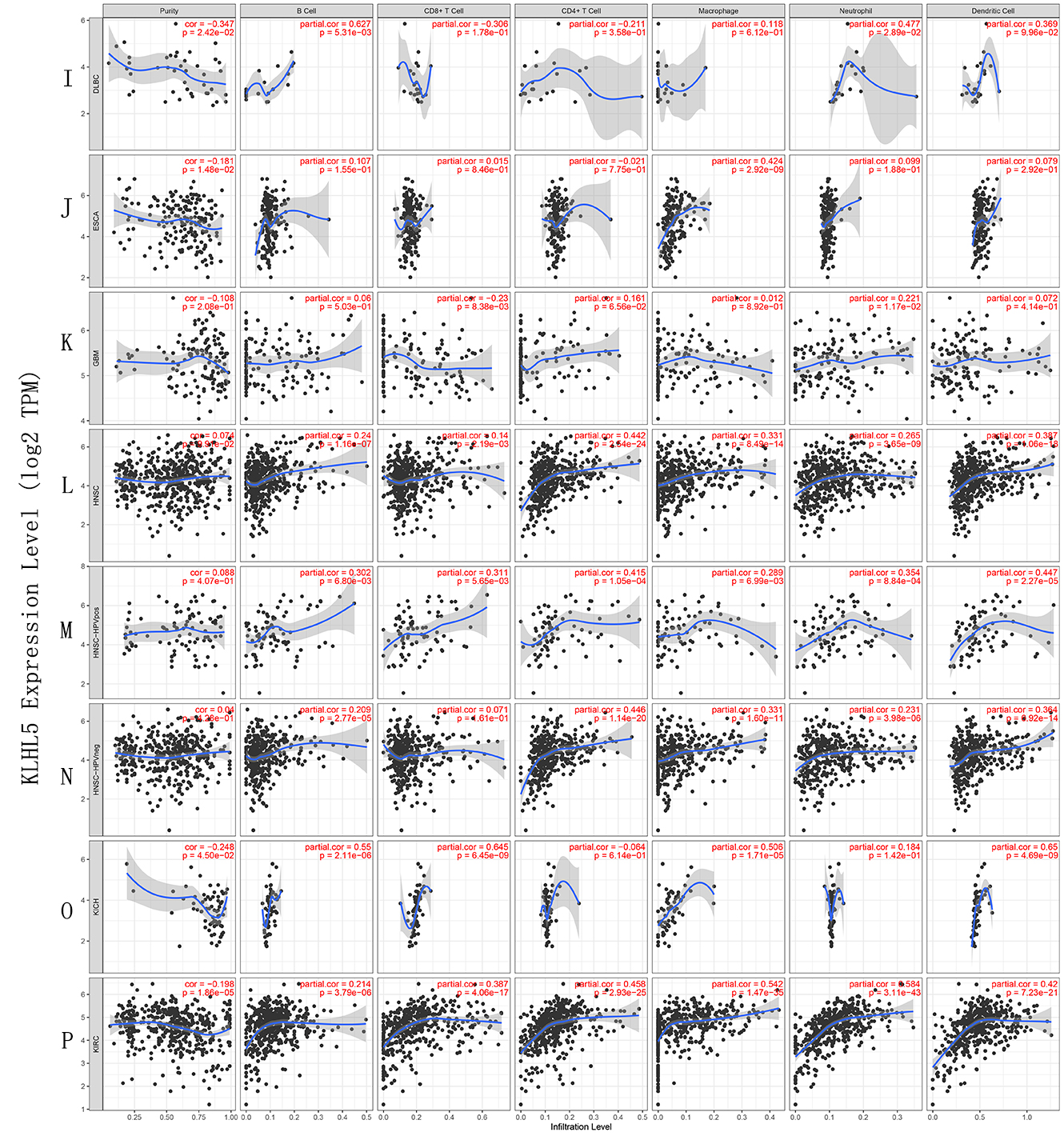

Supplement: Supplementary file 5 [file Image_5.JPEG]

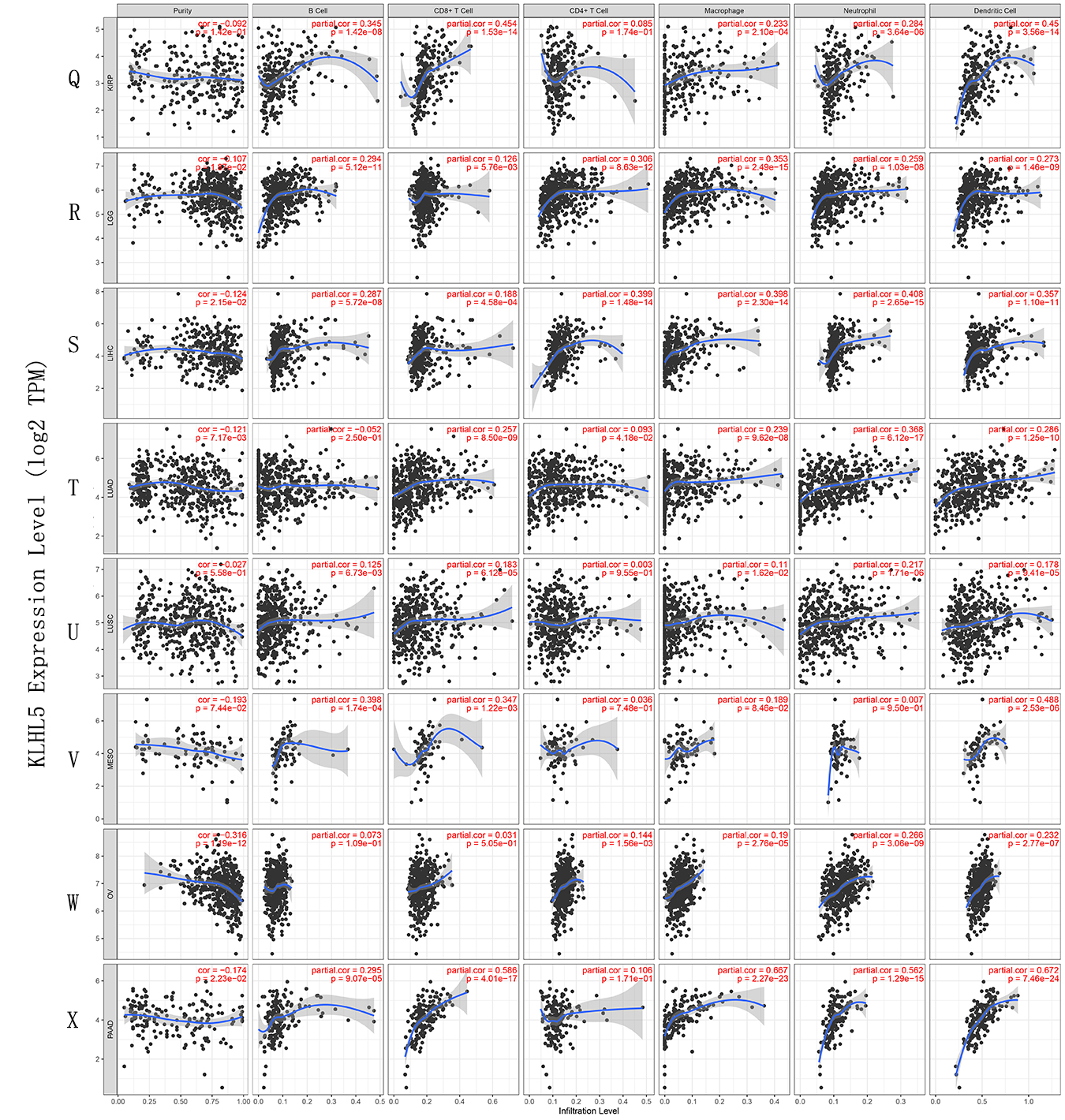

Supplement: Supplementary file 6 [file Image_6.JPEG]

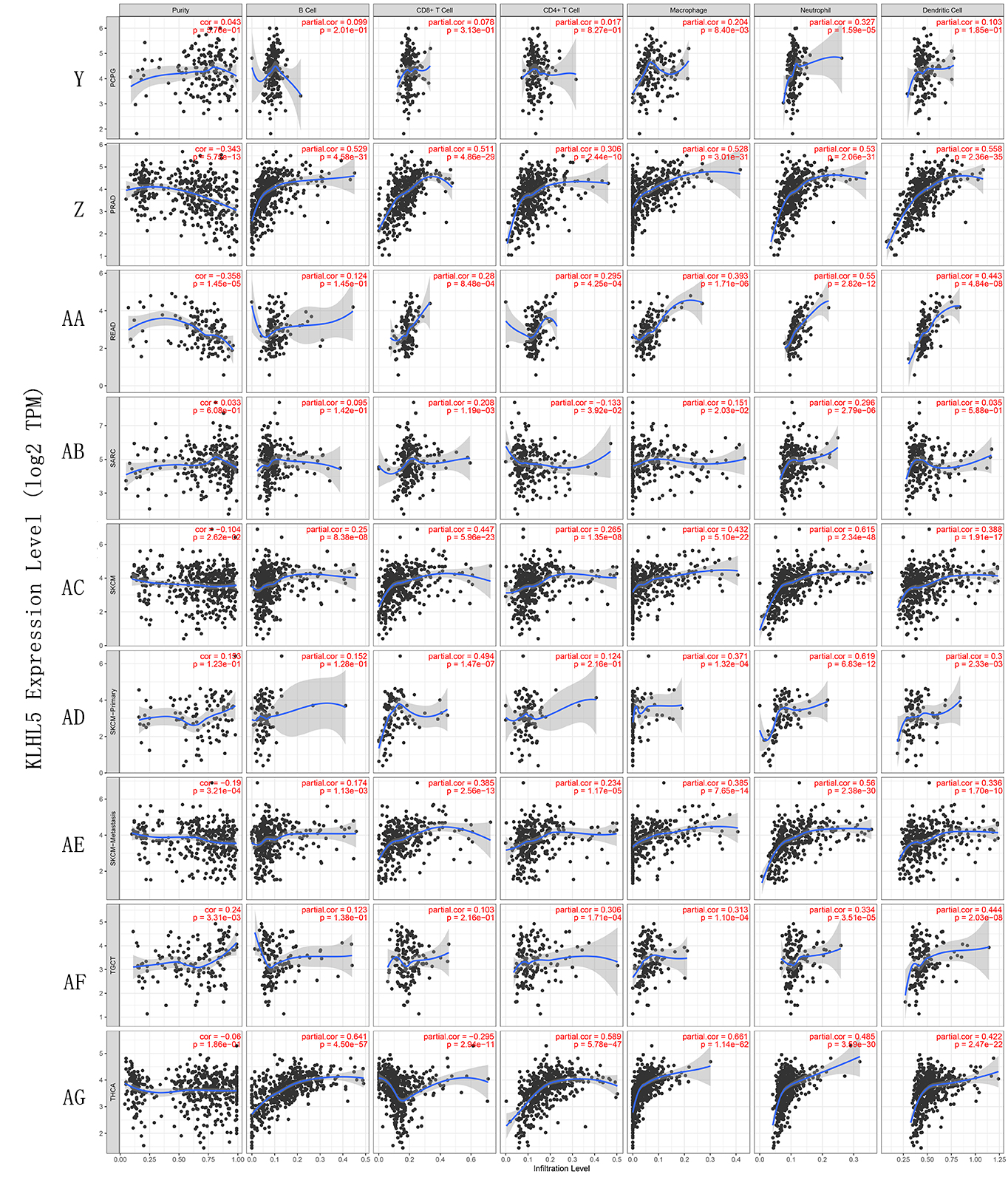

Supplement: Supplementary file 7 [file Image_7.JPEG]

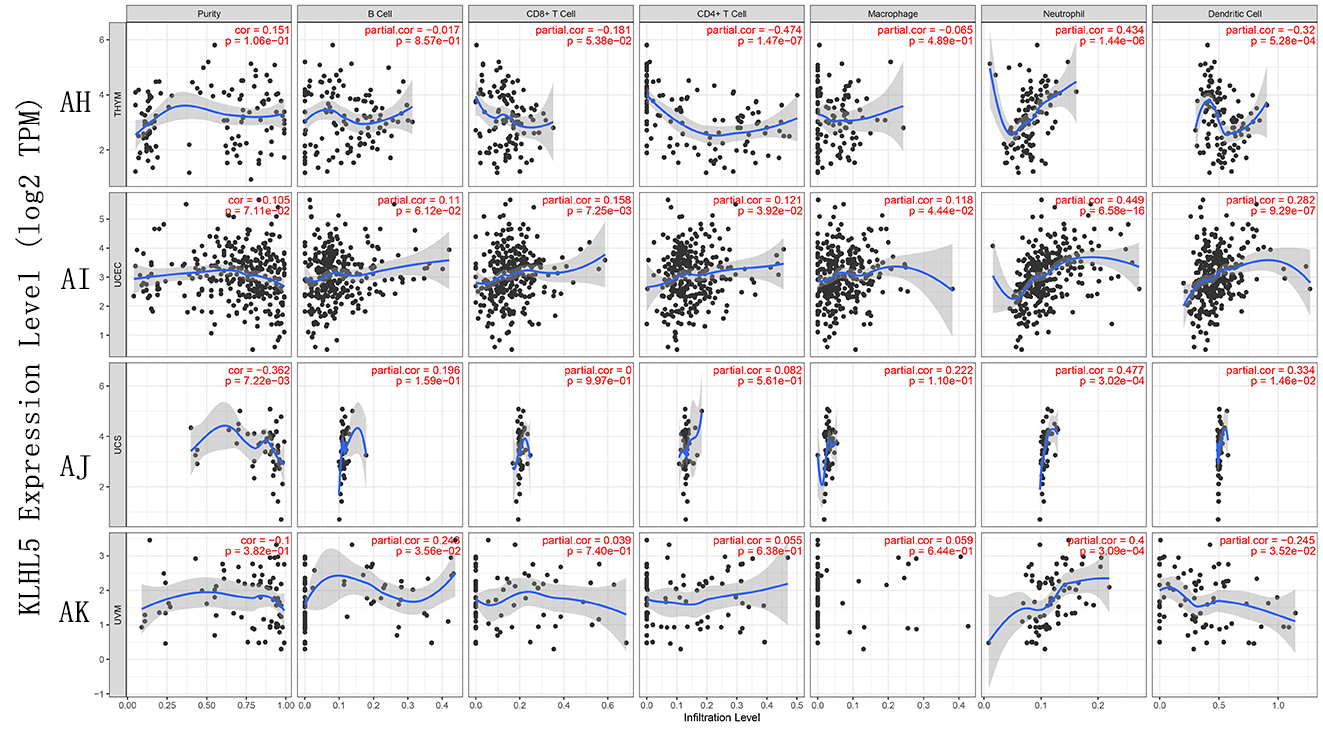

Supplement: Supplementary file 8 [file Image_8.JPEG]
