# Supplementary material for: KLHL5 Is a Prognostic-Related Biomarker and Correlated With Immune Infiltrates in Gastric Cancer
Source: Front Mol Biosci. 2020 Dec 10;7:599110. doi: 10.3389/fmolb.2020.599110 (PMC7758449; doi:10.3389/fmolb.2020.599110)
Supplement: Supplementary file 9 [file Data_Sheet_1.PDF]

Supplementary Table 1 KLHL5 expression in cancers vs normal tissue in Oncomine database

CANCER	CANCER TYPE	P VALUE	FOLD CHANGE	RANK	SAMPLE NUMBER	PMID
Bladder	Infiltrating Bladder Urothelial Carcinoma	0.009	-3.502	5%	47	15930339
	Superficial Bladder Cancer	0.005	-4.454	5%	23	15930339
	Superficial Bladder Cancer	1.12E-10	-2.149	4%	194	20421545
Brain and CNS	Anaplastic Oligoastrocytoma	0.006	2.091	6%	9	16204036
Breast	Invasive Lobular Breast Carcinoma	0.003	2.498	1%	25	17389037
Cervical	Cervical Cancer	1.05E-08	3.545	4%	41	17510386
Colorectal	Colon Carcinoma	2.24E-10	10.705	1%	15	20957034
	Colon Carcinoma	9.54E-09	2.068	3%	15	20957034
Esophageal	Esophageal Adenocarcinoma	4.86E-04	4.875	8%	34	16952561
Gastric	Gastric Mixed Adenocarcinoma	0.001	2.450	10%	35	19081245
Head and neck	Oropharyngeal Carcinoma	1.88E-06	6.747	1%	28	17510386
	Tongue Carcinoma	4.50E-06	2.895	3%	37	17510386
	Floor of the Mouth Carcinoma	1.46E-05	4.448	4%	27	17510386
	Tonsillar Carcinoma	0.005	2.702	6%	28	17510386
	Oral Cavity Carcinoma	0.003	3.321	7%	26	17510386
	Oral Cavity Squamous Cell Carcinoma	1.98E-11	2.691	4%	79	21853135
	Tongue Squamous Cell Carcinoma	3.46E-04	2.371	5%	38	18254958
	Nasopharyngeal Carcinoma	1.05E-04	2.066	7%	41	16912175

Lung	Small Cell Lung Carcinoma	0.007	-2.080	7%	10	11707590
Lymphoma	Angioimmunoblastic T-Cell Lymphoma	2.32E-07	2.084	3%	26	17304354
	Anaplastic Large Cell Lymphoma	5.61E-05	2.999	10%	26	17304354
	Nodular Lymphocyte Predominant Hodgkin's Lymphoma	2.05E-08	-2.07	1%	30	18794340
	Diffuse Large B-Cell Lymphoma	3.26E-09	-2.115	1%	31	18794340
	Diffuse Large B-Cell Lymphoma	6.05E-14	-2.723	4%	64	19412164
	Activated B-Cell-Like Diffuse Large B-Cell Lymphoma	5.23E-08	-4.337	9%	37	19412164
	Anaplastic Large Cell Lymphoma	4.84E-04	-2.466	6%	46	19657361
Myeloma	Monoclonal Gammopathy of Undetermined Significance	4.27E-05	2.195	10%	66	17023574
Others	Mixed Germ Cell Tumor	2.21E-12	2.042	2%	47	16424014
	Teratoma	7.71E-08	2.853	4%	20	16424014
Prostate	Benign Prostatic Hyperplasia Epithelia	7.78E-05	-2.196	1%	12	17173048

Supplementary Table 2 Relation between KLHL5 expression and patient OS in Prognoscan database

CANCER TYPE	DATASET	SAMPLE NUMBER	P-VALUE	HR [95% CI]
Bladder cancer	GSE5287	30	0.460779	0.39 [0.03 - 4.68]
	GSE13507	165	0.835547	0.97 [0.75 - 1.26]
Blood cancer	GSE12417	163	0.728233	1.15 [0.52 - 2.57]
	GSE5122	58	0.76596	0.95 [0.69 - 1.31]
	GSE8970	34	0.883505	0.98 [0.72 - 1.33]
	GSE4475	158	0.00563006	0.20 [0.06 - 0.62]
	GSE16131	180	0.679612	1.05 [0.83 - 1.33]
Brain cancer	GSE4271	77	0.00571792	2.16 [1.25 - 3.73]
	GSE7696	70	0.961758	0.99 [0.65 - 1.50]
	GSE4412	74	0.180535	1.36 [0.87 - 2.12]
	GSE16581	67	0.125482	0.19 [0.02 - 1.58]
Skin cancer	GSE19234	38	0.814227	1.11 [0.46 - 2.71]
Colorectal cancer	GSE17537	55	0.0753153	2.13 [0.93 - 4.92]
	GSE17536	177	0.257654	1.62 [0.70 - 3.72]
Breast cancer	GSE7390	198	0.696077	0.97 [0.81 - 1.15]
Ovarian cancer	GSE26712	185	0.21187	1.78 [0.72 - 4.41]
	GSE17260	110	0.631412	1.11 [0.73 - 1.66]
	GSE9891	278	0.66361	0.91 [0.60 - 1.38]
Lung cancer	GSE13213	117	0.00912698	1.55 [1.12 - 2.16]
	GSE31210	204	0.111192	1.80 [0.87 - 3.71]
	GSE17710	56	0.597087	0.88 [0.56 - 1.40]
	GSE4573	129	0.0173374	1.60 [1.09 - 2.36]
	GSE11117	41	0.249012	0.73 [0.43 - 1.24]
	GSE3141	111	0.946344	0.98 [0.63 - 1.55]

Supplementary Table 3 Relation between KLHL5 expression and patient distant metastasis free survival in Prognoscan database

CANCER TYPE	DATASET	SAMPLE NUMBER	P-VALUE	HR [95% CI]
Breast cancer	GSE19615	115	0.268863	0.48 [0.13 - 1.77]
	GSE6532	87	0.0302665	0.25 [0.07 - 0.88]
	GSE9195	77	0.859612	1.15 [0.24 - 5.52]
	GSE12093	136	0.454768	0.84 [0.53 - 1.32]
	GSE11121	200	0.028946	1.86 [1.07 - 3.25]
	GSE2990	125	0.321115	2.16 [0.47 - 9.86]
Eye cancer	GSE22138	63	0.0930297	0.75 [0.54 - 1.05]

Supplementary Table 4 Relation between KLHL5 expression and patient relapse free survival in Prognoscan database

CANCER TYPE	DATASET	SAMPLE NUMBER	P-VALUE	HR [95% CI]
Breast cancer	GSE9195	77	0.878728	0.94 [0.41 - 2.15]
	GSE12276	204	0.260627	1.22 [0.86 - 1.72]
	GSE9195	77	0.163905	2.76 [0.66 - 11.50]
	GSE6532	87	0.494717	1.37 [0.55 - 3.40]
	GSE2990	125	0.653155	1.31 [0.40 - 4.28]
	GSE7390	198	0.358029	0.94 [0.82 - 1.08]
Head and neck cancer	GSE2837	28	0.402871	0.21 [0.01 - 8.33]
Lung cancer	GSE17710	56	0.610318	1.15 [0.67 - 2.00]
	GSE31210	204	0.0696328	1.67 [0.96 - 2.89]
	GSE8894	138	0.109752	1.41 [0.93 - 2.13]

Supplementary Table 5 Relation between KLHL5 expression and patient disease specific survival in Prognoscan database

CANCER TYPE	DATASET	SAMPLE NUMBER	P-VALUE	HR [95% CI]
Blood cancer	GSE2658	559	0.426415	0.91 [0.71 - 1.16]
Breast cancer	GSE3494	236	0.752425	0.93 [0.60 - 1.44]
	GSE3494	236	0.00156171	0.43 [0.25 - 0.73]
Colorectal cancer	GSE17536	177	0.556075	1.34 [0.51 - 3.55]
	GSE17537	49	0.0205322	3.33 [1.20 - 9.22]
Bladder cancer	GSE13507	165	0.272704	1.21 [0.86 - 1.70]
